# *Plasmodium vivax* Diversity and Population Structure across Four Continents

**DOI:** 10.1371/journal.pntd.0003872

**Published:** 2015-06-30

**Authors:** Cristian Koepfli, Priscila T. Rodrigues, Tiago Antao, Pamela Orjuela-Sánchez, Peter Van den Eede, Dionicia Gamboa, Nguyen van Hong, Jorge Bendezu, Annette Erhart, Céline Barnadas, Arsène Ratsimbasoa, Didier Menard, Carlo Severini, Michela Menegon, Bakri Y. M. Nour, Nadira Karunaweera, Ivo Mueller, Marcelo U. Ferreira, Ingrid Felger

**Affiliations:** 1 Swiss Tropical and Public Health Institute, Basel, Switzerland; 2 University of Basel, Basel, Switzerland; 3 Walter and Eliza Hall Institute, Parkville, Australia; 4 Department of Medical Biology, University of Melbourne, Melbourne, Australia; 5 Department of Parasitology, Institute of Biomedical Sciences, University of São Paulo, São Paulo, Brazil; 6 Department of Vector Biology, Liverpool School of Tropical Medicine, Liverpool, United Kingdom; 7 Department of Biomedical Sciences, Institute of Tropical Medicine, Antwerp, Belgium; 8 Instituto de Medicina Tropical Alexander Von Humboldt, Universidad Peruana Cayetano Heredia, Lima, Peru; 9 National Institute of Malariology, Parasitology, and Entomology, Hanoi, Vietnam; 10 Immunology Unit, Institut Pasteur de Madagascar, Antananarivo, Madagascar; 11 Institut Pasteur de Cambodge, Malaria Molecular Epidemiology Unit, Phnom Penh, Cambodia; 12 Department of Infectious, Parasitic and Immunomediated Diseases, Istituto Superiore di Sanità, Rome, Italy; 13 Department of Parasitology, Blue Nile National Institute for Communicable Diseases, University of Gezira, Wad Medani, Sudan; 14 Department of Parasitology, Faculty of Medicine, University of Colombo, Sri Lanka; 15 Barcelona Centre for International Health Research, Barcelona, Spain; University of California San Diego School of Medicine, UNITED STATES

## Abstract

*Plasmodium vivax* is the geographically most widespread human malaria parasite. To analyze patterns of microsatellite diversity and population structure across countries of different transmission intensity, genotyping data from 11 microsatellite markers was either generated or compiled from 841 isolates from four continents collected in 1999–2008. Diversity was highest in South-East Asia (mean allelic richness 10.0–12.8), intermediate in the South Pacific (8.1–9.9) Madagascar and Sudan (7.9–8.4), and lowest in South America and Central Asia (5.5–7.2). A reduced panel of only 3 markers was sufficient to identify approx. 90% of all haplotypes in South Pacific, African and SE-Asian populations, but only 60–80% in Latin American populations, suggesting that typing of 2–6 markers, depending on the level of endemicity, is sufficient for epidemiological studies. Clustering analysis showed distinct clusters in Peru and Brazil, but little sub-structuring was observed within Africa, SE-Asia or the South Pacific. Isolates from Uzbekistan were exceptional, as a near-clonal parasite population was observed that was clearly separated from all other populations (*F*_ST_>0.2). Outside Central Asia *F*_ST_ values were highest (0.11–0.16) between South American and all other populations, and lowest (0.04–0.07) between populations from South-East Asia and the South Pacific. These comparisons between *P*. *vivax* populations from four continents indicated that not only transmission intensity, but also geographical isolation affect diversity and population structure. However, the high effective population size results in slow changes of these parameters. This persistency must be taken into account when assessing the impact of control programs on the genetic structure of parasite populations.

## Author Summary

*Plasmodium vivax* is the predominant malaria parasite in Latin America, Asia and the South Pacific. Different factors are expected to shape diversity and population structure across continents, e.g. transmission intensity which is much lower in South America as compared to Southeast-Asia and the South Pacific, or geographical isolation of *P*. *vivax* populations in the South Pacific. We have compiled data from 841 isolates from South and Central America, Africa, Central Asia, Southeast-Asia and the South Pacific typed with a panel of 11 microsatellite markers. Diversity was highest in Southeast-Asia, where transmission is intermediate-high and migration of infected hosts is high, and lowest in South America and Central Asia where malaria transmission is low and focal. Reducing the panel of microsatellites showed that 2–6 markers are sufficient for genotyping for most drug trials and epidemiological studies, as these markers can identify >90% of all haplotypes. Parasites clustered according to continental origin, with high population differentiation between South American and Central Asian populations and the other populations, and lowest differences between Southeast-Asia and the South Pacific. Current attempts to reduce malaria transmission might change this pattern, but only after transmission is reduced for an extended period of time.

## Introduction

*Plasmodium vivax* is the human malaria parasite with the largest geographical expansion, and the predominant malaria parasite outside of Africa [1]. Transmission intensity (according to annual parasite incidence as a surrogate measure) ranges from very low and seasonal in temperate zones and in countries approaching malaria elimination to very high mainly in Asian and South Pacific countries [1]. Prior to malaria control starting early in the 20^th^ century, *P*. *vivax* transmission even occurred in large parts of Europe, Russia and the US [2]. *P*. *vivax* is difficult to control due to relapsing liver stages, fast and constant formation of gametocytes and a large proportion of asymptomatic carriers contributing to transmission [3, 4]. As a consequence, *P*. *vivax* has become the predominant malaria parasite in several countries where *P*. *falciparum* transmission has been successfully reduced [5, 6].

Along with other parameters, parasite diversity can be used to assess the effect of interventions, as reduced transmission is expected to result in reduced diversity. This relationship was observed for *P*. *falciparum* [7, 8]. Moreover, knowledge on parasite diversity is the basis to study gene flow between populations, or to track the source of imported infections [9]. Thus, global comparisons of population genetic data help to develop and validate molecular tools for surveillance of antimalarial interventions.

Typing of highly polymorphic microsatellites has proven useful to describe the diversity and structure of parasite populations [10–14], to study patterns of relapses [15–17], multiplicity and molecular force of infection [18, 19], and for distinguishing reinfection from recrudescence in drug trials [20–22]. Several studies reported extensive *P*. *vivax* microsatellite diversity even in regions of moderate endemicity, and high multiplicity of infection was frequently observed. While some *P*. *vivax* populations showed pronounced structuring on small geographical scale [10, 13], this was not the case for other populations [11, 23].

Differences in method of sampling with respect to geographical space as well as different panels of markers used for genotyping make direct comparison of results difficult [24]. Lacking so far was a comprehensive comparison of *P*. *vivax* diversity based on samples collected across many different sites and typed with the same set of markers. Therefore we compiled data from published studies that included samples from Peru [10], Brazil [14, 21], Sudan [25], Cambodia [26], Vietnam [27], Papua New Guinea (PNG) and Solomon Islands [11], and complemented this dataset with previously unpublished typing results from Central America and Mexico, Madagascar and Central Asia (Armenia, Azerbaijan and Uzbekistan). All samples were typed with 11 published microsatellite markers [28].

**Fig 1:**
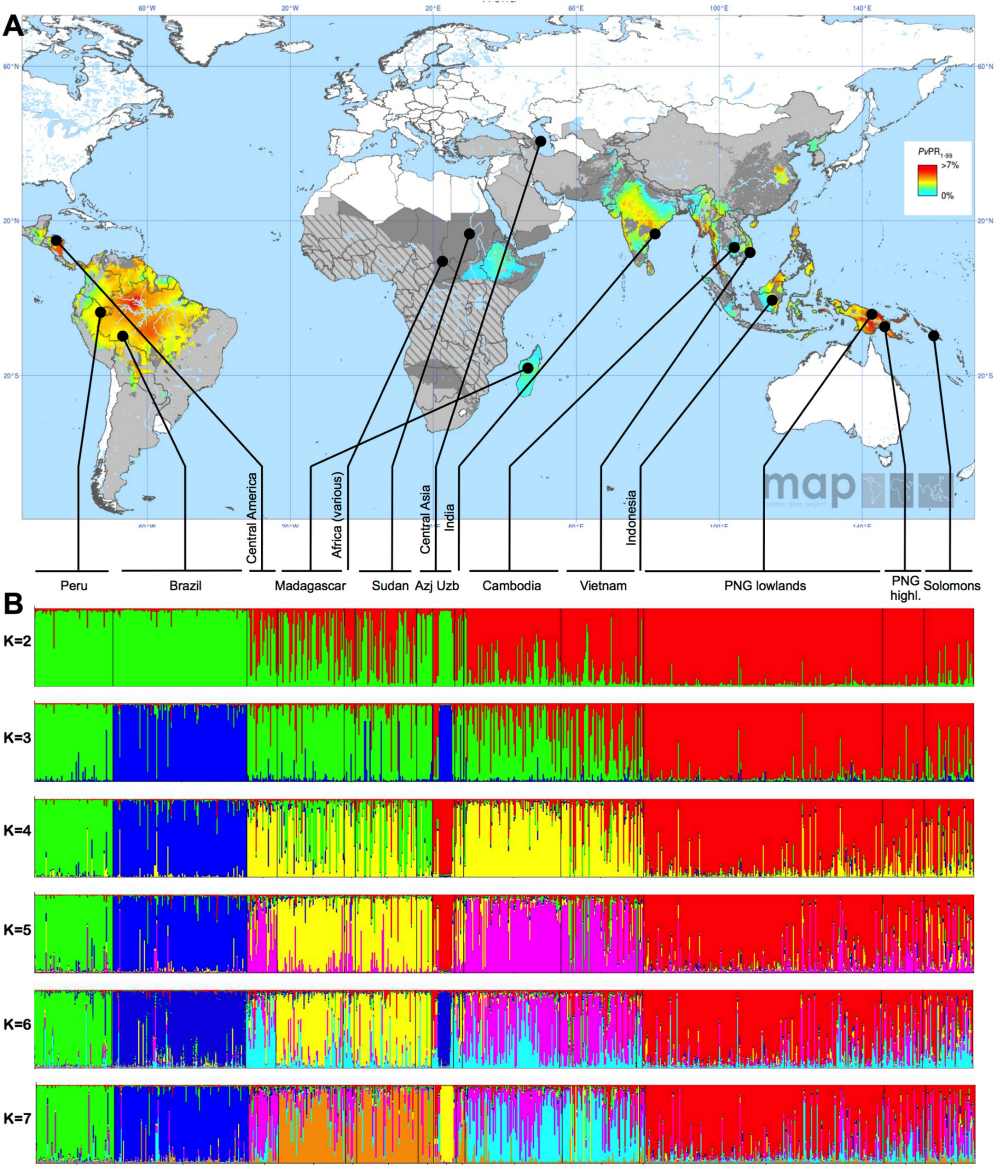
Origin of parasite isolates and cluster analysis. (A) The collection sites of the parasite isolates are indicated on a map of *P*. *vivax* endemicity in 2010 [1]. Note that populations named ‘Central America’, ‘Africa’, ‘Central Asia’, ‘India’ and ‘Indonesia’ include isolates from various locations within these regions (B) Cluster analysis for K = 2 to K7. Azj = Azerbaijan, Uzb = Uzbekistan

This global data set included 841 isolates from regions of different levels of transmission intensity (Fig 1A and Table 1) and representing 6 out of 9 malaria transmission zones recently shown to differ in relapse patterns [29]. The highest *P*. *vivax* prevalence ever has been recorded in the lowlands of PNG (e.g. reaching >50% by PCR in children in East Sepik Province [19]). These South Pacific parasite populations are relatively isolated due to limited migration of human hosts. In Southeast (SE)-Asia transmission is also high, yet often focal [30]. Migration of hosts is high SE-Asia and a major complication of eradication efforts. In Latin America transmission is lower, but increased since the 1960s when the number of *P*. *vivax* cases was very low due to successful spraying campaigns [5]. Transmission in Central America is low, and parasite populations are separated from those in South America by the Isthmus of Panama, with no road connecting South and Central America. Also within Central America sub-structuring is likely; e.g. different *P*. *vivax* subpopulations were observed in Mexico, following vector distribution [31]. In Africa, *P*. *vivax* transmission occurs mostly in Madagascar and East Africa, i.e. Ethiopia and Sudan. In other parts of Sub-Saharan Africa *P*. *vivax* transmission is very low as most individuals carry the Duffy-negative blood type, which largely prevents *P*. *vivax* infection [32]. Thus, Madagascan parasites are isolated from other *P*. *vivax* populations in northern Africa, and transmission in Madagascar is relatively low. In Central Asia, transmission is very low; in Uzbekistan malaria had been eradicated in 1961, but was reintroduced later, and is characterized by small outbreaks in border areas [33].

**Table 1:** Origin of samples included in study.

Population	n	Year	Details of location	Details of sample collection	Published	References
Peru	70	2006–2008	Several villages around Iquitos, Peruvian Amazon	Febrile patients	Yes	[10]
Brazil	120	2004–2005 /2006	Granada farming settlement, next to Acrelândia, Acre State, approx. 50 km from the border with Bolivia	Febrile patients	Yes	[72]
Central America and Mexico	27	2004–2008	Different Central American countries and Mexico	Travelers returning to the USA	Yes	[38]
Madagascar	60	2006–2008	Miandrivazo and Tsiroanomandidy, approx. 100 km apart on the central highland plateau	Febrile patients	No	[79]
Africa	9	2004–2008	5 isolates from Ethiopia, 4 from across sub-Saharan Africa	Travelers returning to the USA	Yes	[38]
Sudan	55	2007	Wad Medani–Gezira State (Central Sudan) and New Halfa–Kassala State (East Sudan).	Febrile patients	Partly	[25]
Armenia / Azerbaijan	14	2001–2003		Febrile patients	Partly	[25]
Uzbekistan	20	2001–2003		Febrile patients	No	
India	8	2004–2008	India + 1 isolate from Bangladesh	Travelers returning to the USA	Yes	[38]
Cambodia	87	2008	Pursat town	Febrile patients	Yes	[26]
Vietnam	69	1999–2000	Binh Thuan Province, central Vietnam	Febrile patients identified through active case detection	Yes	[27, 30]
Indonesia	5	2004–2008		Travelers returning to the USA	Yes	[38]
PNG lowlands	214	2003–2007	North coast of PNG: Ilaita + Kunjingini (East Sepik Province), Alexishafen (Madang province)	Ilaita: cohort study, Kunjingini / Alexishafen: febrile children	Yes	[11]
PNG highlands	37	2004–2005	Sigimaru, southern highlands fringe	Febrile children	Yes	[11]
Solomon Islands	45	2004–2005	Tetere, Guadalcanal Province	cross-sectional survey	Yes	[11]

The wide distribution of parasite populations included in this study permit for the first time assessing *P*. *vivax* microsatellite population structure on a global scale. Previous studies on *P*. *vivax* population structure across different continents mostly genotyped polymorphic antigens [34, 35] or mitochondrial DNA [36, 37]. These markers differ from microsatellites because antigens are under immune selection and mitochondria are maternally inherited, thus excluding recombination. It remains unclear whether similar results would be obtained from analyses of mtDNA, antigen-coding genes or of putatively neutral microsatellites. The present study allowed a global comparison of parasite microsatellite diversity and population structure made possible by harmonization of methods, and the definition of a minimal subset of markers for epidemiological studies.

## Methods

### Ethics statement

Informed written consent was obtained from all individuals or their parents or guardians prior to the study. Details on ethical approval are published for samples from Peru [10], Brazil [14, 21], Sudan [25], Vietnam [27], Cambodia [26] and the South Pacific [11]. For samples collected from returning travelers to the US (samples from Central America, Mexico, Africa, India and Indonesia) the study protocol was approved by the Ethical Committee for Research with Human Subjects of the Institute of Biomedical Sciences, University of São Paulo (960/CEP). In Madagascar, the study protocol was approved by the Ethics Committee of the Ministry of Health of Madagascar (007/SANPF/2007). The study was approved by the WEHI Human Research Ethics Committee.

### Study sites and sample collection

Details of samples included in this study are given in Table 1. In case of cohort studies, only 1 sample per individual was included. From Africa, India and Indonesia samples from returning travelers were utilized [38]. While the number of travelers’ samples was too small to assess intra-population diversity, linkage disequilibrium, or *F*_ST_ compared to other populations, they were still useful for clustering analysis and principal component analysis (PCA).

Samples from Central America and Mexico had been collected from travelers returning from several countries spanning from Panama to Mexico. Due to the limited number of isolates and lack of precise information on sample origin (some samples derived from travelers visiting several countries), these isolates were combined as ‘Central American’ population, despite possible sub-structuring. From Armenia and Azerbaijan 14 isolates were available; these were combined because migration between both countries is frequent and it was not always clear in which of the two countries the infection had been acquired. From Uzbekistan 20 isolates were available. For calculation of allelic richness all Central Asian countries were pooled to reach the required number of 25 samples.

### Genotyping and data analysis

All samples were typed with the same set of 11 published microsatellite markers [28]. Three additional markers of that panel were excluded. MS3 and MS16 had not been typed in all populations, and MS8 showed signals of positive selection and variation in allele sizing between labs (details below). Microsatellites are considered neutral markers; however, some of them might lie within coding regions or be linked to genes under selection. Selection had been tested using Lositan software [39]. Only marker MS8 showed a weak signal for positive selection. A relationship between *P*. *vivax* microsatellite alleles and clinical disease or acquired immunity has never been reported, thus it was not to be expected that different age groups sampled across populations or different proportions of febrile and asymptomatic individuals would influence population genetic parameters.

Minor differences in typing protocols did not affect the results; i.e. samples from PNG, Solomon Islands, Madagascar Sudan and Central Asia were amplified by nested PCR, while a single round of PCR was performed on all other samples. As the nested PCR primers were identical to the single-round primers, allele sizes can be compared directly. From each lab a subset of samples was typed again starting from DNA to ensure comparability of results. Allele lengths were very consistent for all markers, except for MS8, where variation of up to 1.5 base pairs was observed. Due to this variation and possible positive selection MS8 was excluded from analysis.

In case of multi-clone infections the predominant peak only was included into the analysis. Occasionally this can result in incorrect haplotype assembly. This affects analysis such as linkage disequilibrium or clustering, where individual haplotypes are needed. Diversity and *F*_ST_ values are not affected because allelic frequencies are assessed at population level. Clustering analysis and calculation of LD were repeated with only those samples that harbored a single allele at each marker. To obtain sufficient samples from Madagascar and Cambodia, we permitted in the analysis also isolates with >1 alleles at one of the markers. It should be noted that isolates of low multiplicity were selected for genotyping for several parasite populations. Thus the difference between the number of samples of the full data set (including predominant peak haplotypes) and the number of only single clone infections does not reflect the proportion of multiple clone infections.

Alleles were binned using TANDEM software [40] and formatted using PGDspider [41]. Expected heterozygosity (*H*_E_) of markers and allelic richness were calculated using FSTAT [42]. *H*_E_ is the chance that two unrelated parasites carry a different allele of a given marker, and allelic richness is a measure of alleles per each marker adjusted for the different numbers of isolates per site. When the effective population size is reduced, e.g. as consequence of intensified malaria control, rare alleles are expected to disappear first. As a result, allelic richness changes more rapidly than *H*_E_, as the influence of low-frequency alleles on *H*_E_ is small. The number of unique haplotypes was calculated by Dropout [43] and linkage disequilibrium (LD) by LIAN 3.5 with 100,000-fold re-sampling [44]. LIAN compares the observed association of markers to the values expected for random association based on the population diversity. Only unique haplotypes with no missing data were included for calculating LD, resulting in 633 samples in this analysis. Three of the markers used (MS2, MS4, MS5) localize to chromosome 6 and two markers (MS12, MS15) to chromosome 5, thus these markers are physically linked. To assess linkage disequilibrium irrespective of physical linkage of markers, LD was also calculated excluding markers MS2, MS4 and MS12, i.e. with 8 markers located on 8 different chromosomes. As compared to the 11-marker panel, fewer isolates had to be excluded due to missing data, but identical 8-marker haplotypes occurred more often, resulting in 637 samples for this analysis.

Relatedness between haplotypes was assessed by pairwise comparison of all samples within a population and calculating the proportion of shared alleles. Only samples with at least 7 markers available for comparison were included.

Effective population size *N*_e_ (i.e. the estimated number of unique haplotypes circulating in each site) was calculated using step-wise mutations models (SMM) as well as infinite allele models (IAM), using mutation rates observed in *P*. *falciparum* studies of 1.59*10^−4^ (95% confidence interval = 3.7*10^−4^, 6.98*10^−5^) [45]. While some of the markers harbor simple tri-nucleotide repeats, and SMM are likely applicable, other markers contain more complex repeat structures (e.g. MS2, MS6, MS10, MS20) and IAM are more appropriate [46], thus both values are given.

The software STRUCTURE was used to assess clustering of isolates [47]. This method detects clusters without prior information on the origin of samples. Twenty iterations for K = 1 to K = 12 (K being the number of clusters) were run, each with a burn-in period of 10’000 steps and then 100’000 MCMC iterations. A method developed through simulation studies [48] was applied to estimate the most likely number of clusters. In addition, the optimal number of clusters was assessed using the program STRUCTURAMA [49]. *F*_ST_ values among populations were calculated using FSTAT [42]. To compute Principal Components Analysis (PCA) the smartPCA application of EIGENSOFT was used [50]. In contrast to STRUCTURE analysis, PCA attempts to maximize variance between populations based on the known origin of samples. While smartPCA was designed for SNPs it can be used with microsatellites; each microsatellite allele was treated as a SNP. Preliminary analysis had shown no population substructuring between samples collected in Brazil in 2004 and 2006 [21], in the lowlands of PNG [11] and in Madagascar, thus samples were combined for calculation of *F*_ST_ values and PCA.

**Fig 2:**
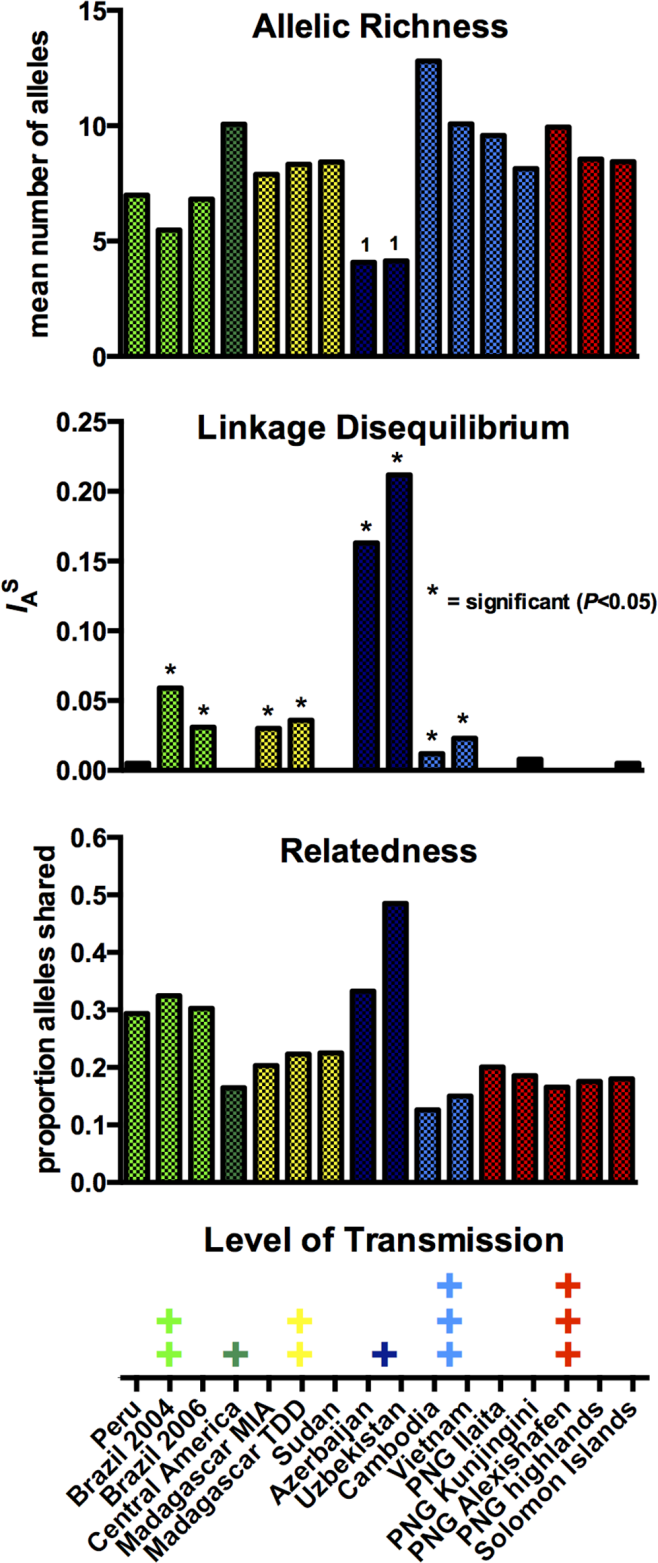
Within-population diversity, LD and haplotype relatedness in relation to transmission intensity. Transmission intensity is given in levels, low, medium and high. Diversity is given as mean allelic richness of 11 markers, based on 25 individuals (^1^ as for Azerbaijan and Uzbekistan few isolates were available, allelic richness is based on 12 individuals). For LD the standardized index of association (*I*_A_^S^) of given, including only 1 marker per chromosome, and excluding identical haplotypes within populations. Relatedness shows the mean number of alleles shared for any pair-wise comparison within a population.

In the absence of the same measures of transmission intensity for all populations, such as entomological inoculation rate, force of infection or parasite prevalence, populations were broadly classified as low, medium and high transmission (Fig 2).

## Results

A total of 841 *P*. *vivax* isolates were available for this study. Fig 1A shows the origin of isolates on a published map of *P*. *vivax* endemicity in 2010, represented as parasite rate [1]. All isolates were typed for the same set of 11 microsatellite markers. For all 841 isolates genotyping results were obtained from ≥7 markers. For marker MS4, no amplification product was obtained from 70 isolates (i.e. data was obtained from 91.7% of all isolates), for all other markers data was obtained from 823–834 (97.9–99.2%) out of 841 isolates.

### Genetic diversity, linkage disequilibrium and effective population size

Pronounced differences in population diversity were observed. While expected heterozygosity (*H*_*E*_) was generally high, it was strongly reduced in isolates from Uzbekistan. With the exception of Uzbekistan, *H*_E_ of all except 2 markers (MS5 and MS7) was >0.5 in all populations (Table 2, supplementary S1 File). Mean *H*_*E*_ of 11 markers was lowest in Uzbekistan (0.52), followed by Azerbaijan (0.67) and South America (0.68–0.71), intermediate in Africa and the South Pacific (0.77–0.83), and highest in South-East Asia (0.84–0.87). The differences between continents were slightly higher when only those markers with lower overall diversity were assessed (MS1, MS4, MS5, MS7, MS12, mean *H*_*E*_<0.75). Mean *H*_*E*_ ranged from 0.53 in Uzbekistan and 0.6 in Peru to 0.85 in Cambodia. Mean allelic richness showed a similar pattern (Table 2 and Fig 2) with lowest values in South America (5.5–7.0 alleles/locus) and Central Asia (7.1 alleles/locus), intermediate values in Africa and the South Pacific (7.9–9.9 alleles/locus), and highest in Cambodia (13.4 alleles/locus).

**Table 2:** Diversity of 11 microsatellite loci.

Population	Sub-population	N	Mean *H*_E_ 11 markers	Mean *H*_E_ 5 markers (*H*_E_ <0.8)^1^	Mean number of alleles	Mean allelic Richness^2^
South America	Combined	190	0.79	0.72	11.73	8.99
Peru		70	0.71	0.6	7.64	6.99
Brazil	Combined	120	0.74	0.7	9.27	7.21
	Brazil 2004	42	0.68	0.63	5.73	5.48
	Brazil 2006	78	0.70	0.69	8.09	6.82
Central America and Mexico		27	0.84	0.78	10.09	10.07
Madagascar	Combined	60	0.80	0.74	10.09	9.10
	Miandrivazo	28	0.80	0.73	7.91	7.89
	Tsiroano-mandidy	32	0.78	0.72	8.45	8.33
Sudan		55	0.76	0.71	9.36	8.43
Central Asia	Combined	34	0.75	0.74	7.27	7.16
	Azerbaijan	14	0.67	0.66	4.09	NA^3^
	Uzbekistan	20	0.52	0.54	4.36	NA^3^
Cambodia		87	0.87	0.85	16.18	12.81
Vietnam		69	0.84	0.8	11.45	10.08
PNG lowlands	Combined	214	0.80	0.74	14.91	10.15
	Iliata	132	0.79	0.7	12.55	9.58
	Kunjingini	38	0.81	0.72	8.36	8.15
	Alexishafen	44	0.83	0.77	10.64	9.94
PNG highlands		37	0.82	0.78	8.82	8.56
Solomon Islands		46	0.81	0.77	8.91	8.44

Detailed data for all markers and all populations can be found in Supplementary S1 File.

^1^ Includes markers MS1, MS4, MS5, MS7, MS12

^2^ Mean allelic richness is based on 25 individuals

^3^ Not available, as number of samples <25

When all isolates and all markers were analyzed, linkage disequilibrium (LD) was strong and significant in South American, Madagascan, Central Asian and SE-Asian populations (Table 3). No or limited LD was detected in Central America, Sudan and the South Pacific. Trends were similar when only single-clone infections were analyzed, with exception of Peru and Madagascar, where LD observed in the analysis of all data was no longer detected. Overall levels of LD were lower in the single-clone data set. This can be explained by the reduction in sample size, since similarly low levels of LD were observed in an equally low number of randomly selected multi-clone infections. Most representative results were obtained when only 1 marker per chromosome was included, thus excluding physical linkage of markers (total of 8 markers). All populations in the South Pacific, Peru, Central America and Sudan were in full linkage equilibrium (Table 3 and Fig 2).

**Table 3:** Linkage disequilibrium.

Population	Sub-population	All isolates			Single-clone infections			1 marker/chromosome^1^		
		n^2^	*I*_A_^S^	*P*	n^2^	*I*_A_^S^	*P*	n^2^	*I*_A_^S^	*P*
Peru		52	0.032	2.1 x 10^−4^	17	0.007	0.31	44	0.005	0.33
Brazil	Combined^4^	111	0.069	<10^−5^	32	0.036	<10^−5^	81	0.028	<10^−5^
	Brazil 2004	35	0.130	<10^−5^	14	0.090	1.1 x 10^−4^	26	0.059	1.3 x 10^−4^
	Brazil 2006	76	0.071	<10^−5^	18	0.088	<10^−5^	58	0.031	10^−4^
Central America and Mexico		27	0.020	0.027	NA			27	-0.005	0.62
Madagascar	Combined^5^	53	0.033	<10^−5^	12	-0.008	0.67	52	0.025	3 x 10^−4^
	Miandrivazo	24	0.057	<10^−5^	NA			24	0.030	0.016
	Tsiroanomandidy	29	0.062	<10^−5^	NA			30	0.036	1.5 x 10^−3^
Sudan		36	0.013	0.051	18	0.001	0.48	38	-0.011	0.89
Central Asia		10	0.203	<10^−5^	9	0.179	<10^−5^	15	0.163	<10^−5^
Cambodia		87	0.011	4.3 x 10^−4^	17	0.035	0.013	86	0.012	2.1 x 10^−3^
Vietnam		43	0.078	<10^−5^	NA			44	0.023	1.1 x 10^−3^
PNG lowlands	Combined^6^	163	0.003	0.085	67	0.001	0.43	184	-0.001	0.68
	Iliata	91	0.000	0.49	28	0.001	0.43	110	0.000	0.5
	Kunjingini	31	0.011	0.047	14	0.033	0.028	36	0.008	0.14
	Alexishafen	41	0.010	0.036	25	0.000	0.47	41	-0.002	0.59
PNG highlands		29	0.017	0.015	8	0.036	0.11	30	-0.009	0.85
Solomon Islands		22	0.002	0.44	NA			36	0.005	0.26

^1^ includes 8 microsatellite markers located on 8 different chromosomes

^2^ n = number of unique isolates (no identical haplotype in the same population) without missing data; this was the data analyzed

Effective population *N*_e_ size was about twice as high in Cambodia and Vietnam (IAM: 6378 and 5553) as compared to South America (2423–2829, Table 4). Values for Madagascar, Sudan and the South Pacific were intermediate, and low for Azerbaijan (2422) and Uzbekistan (1606). Estimates based on SMM were 2–3 fold higher. Samples from Central America and Mexico were highly diverse and showed high *N*_e_ (5159), likely because they originated from different countries and thus represent different subpopulations.

**Table 4:** Effective population size.

Population	Sub-population	IAM	SMM
Peru		2829 [1216–6443]	5373 [2309–12239]
Brazil	Combined	3429 [1473–7810]	7167 [3080–16326]
	Brazil 2004	2423 [1041–5519]	4289 [1843–9771]
	Brazil 2006	2598 [1116–5917]	4743 [2038–10805]
Central America and Mexico		5159 [2217–11753]	13625 [5855–31036]
Madagascar	Combined	4373 [1879–9961]	10453 [4492–23812]
	Miandrivazo	4327 [1859–9856]	10280 [4418–23418]
	Tsiroanomandidy	3837 [1649–8739]	8517 [3660–19401]
Sudan		4829 [2075–11000]	12245 [5262–27894]
Azerbaijan		2422 [1041–5517]	4287 [1842–9766]
Uzbekistan		1606 [690–3658]	2426 [1043–5527]
Cambodia		6378 [2741–14529]	19315 [8300–43999]
Vietnam		5553 [2386–12649]	15359 [6600–34987]
PNG lowlands	Combined	4626 [1988–10539]	11433 [4913–26044]
	Iliata	4192 [1801–9549]	9780 [4203–22277]
	Kunjingini	4659 [2002–10614]	11563 [4969–26340]
	Alexishafen	5092 [2188–11598]	13336 [5731–30378]
PNG highlands		5010 [2153–11412]	12991 [5583–29593]
Solomon Islands		4675 [2009–10649]	11624 [4995–26480]

### Haplotype diversity

Across all populations a total of 759 individual haplotypes were found in 818 isolates. 11-loci haplotypes were shared only within the same country. Again isolates from Uzbekistan were unusual, as 10 out of 20 isolates were identical and another two shared the same haplotype. Ten haplotypes were observed more than once in Peru, 5 and 2 in Brazil in 2004 and 2006 (plus 1 shared between the 2004 and 2006 data set), 5 in Sudan, 2 in Azerbaijan, 2 in Vietnam (1 observed 5 times), and 1 in PNG. 730/818 haplotypes were singletons.

Relatedness among haplotypes was calculated for each population (Fig 2). In the pairwise comparisons isolates from Cambodia and Vietnam shared the same allele on average in 12.6% and 15.0% of markers (i.e. a mean of 1.4 and 1.65 out of 11 microsatellites carried the same allele). Samples from Central American, African and South Pacific populations shared the same allele in 16.5–22.5% of markers. Relatedness was 29.3–32.5% in South American populations, and in Azerbaijan 33.3% and in Uzbekistan 48.5% of markers shared the same allele.

**Fig 3:**
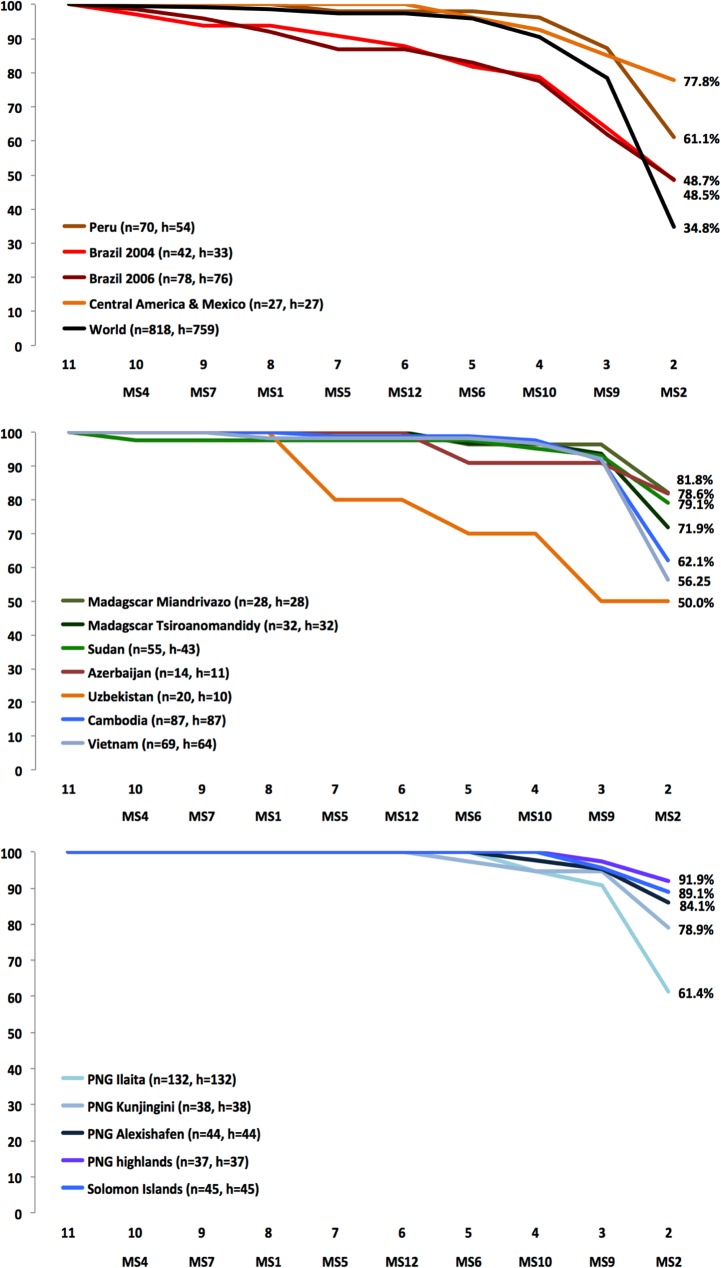
Loss of discrimination power between haplotypes with a reduced panel of markers. Markers were sequentially removed (X-axis: number of remaining marker and marker removed), and the percentage of individual haplotypes as compared to the 11-marker panel was counted. n = number of isolates per population, h = number of individual haplotypes identified with the 11-marker panel. ‘World’ shows the number of haplotypes when those shared between populations were counted only once.

To assess discrimination power of a smaller panel of markers compared to the full set of markers, microsatellites were removed successively and haplotype counts recorded from each individual population, as well as for the full dataset (Fig 3). MS4 was removed first, as no data for this marker could be obtained from 70 isolates. The remaining 10 markers were sequentially removed according to their diversity across all populations, starting with the least diverse one.

Only taking into account haplotypes shared within populations, a set of 4 markers (MS2, MS9, MS15, MS20) identified 710/760 (93.4%) haplotypes and 3 markers (MS2, MS15, MS20) still identified 665/760 (87.5%) haplotypes. By reducing to 2 markers (i.e. MS15 and MS10), the number of haplotypes dropped considerably to 513/760 (67.5%). The proportion of haplotypes lost by omitting markers was highest in South American populations. When using only 3 markers, 13–38% of haplotypes were lost in South American populations, but <10% in populations from SE-Asia and the South Pacific (Fig 3).

Taking into account haplotypes observed in several populations, a single haplotype was shared between Peru and Vietnam when the panel was reduced to 5 markers. With four markers 15 additional haplotypes were shared. While some were shared between populations from the same continent (5 identical 4-loci haplotypes in 251 isolates from PNG), 11 haplotypes occurred on different continents (e.g. Peru/Azerbaijan, Madagascar/Vietnam, Brazil/Cambodia/Vietnam, Sudan/Solomon Islands). As a consequence, when haplotypes shared between populations were taken into account, 4 markers identified 687/759 (91.5%) haplotypes, and 3 markers 595/759 (78.4%) haplotypes (black line in Fig 3, panel 1).

### Population structure

**Fig 4:**
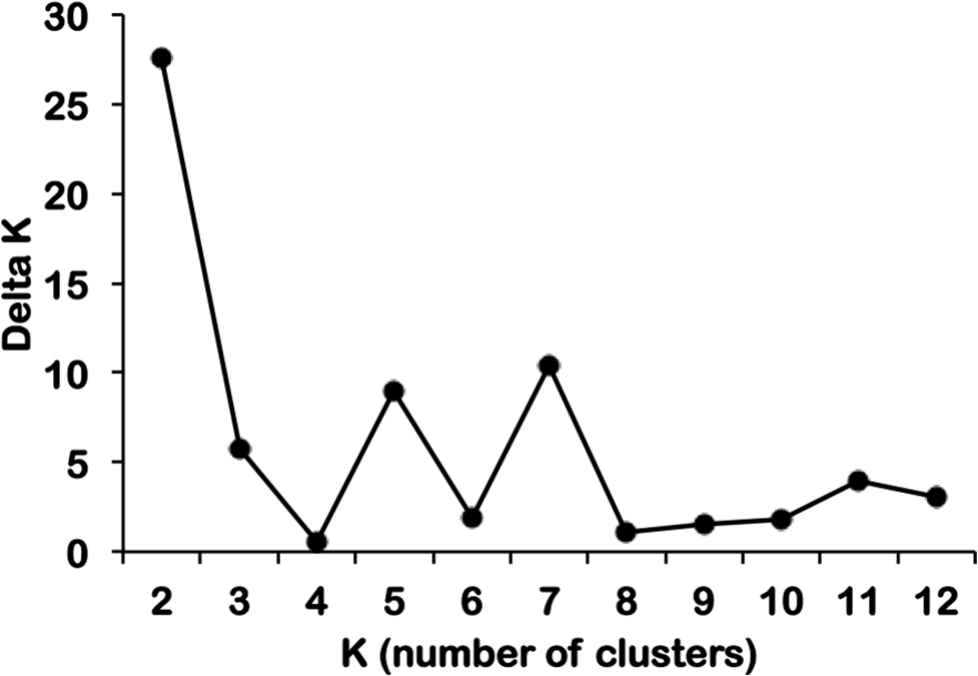
Optimal number of clusters. The optimal number of clusters for the STRUCTURE analysis shown in Fig 1 was calculated as described [48].

Clustering analysis indicated clearly distinct clusters, mainly following geographical lines (Fig 1B). First South American samples (but not those from Central America) and samples from Azerbaijan were separated from all other populations, with admixture in samples from Central America and Africa. When the number of clusters (K) was 3, the South Pacific populations formed a separate cluster, as well as the Brazilian samples, while samples from Peru, Central America, Madagascar and Asia clustered together. Peruvian samples formed a separate cluster when K was set to 4, K = 5 led to the separation of Madagascar, Sudan and Azerbaijan from SE-Asia. Central American samples formed an individual cluster when K = 6. The clonal population in Uzbekistan clustered with different populations in individual STRUCTURE runs for a given value of K, indicating that no clear relationship to any other population was observed. When the optimal number of clusters was calculated as described [48], high values were observed for K = 2, K = 5 and K = 7 (Fig 4). In addition the program STRUCTURAMA was used to assess the best number of clusters. The 99% CI for estimates of cluster number showed a wide range (79–115 clusters) and was thus not informative

**Fig 5:**
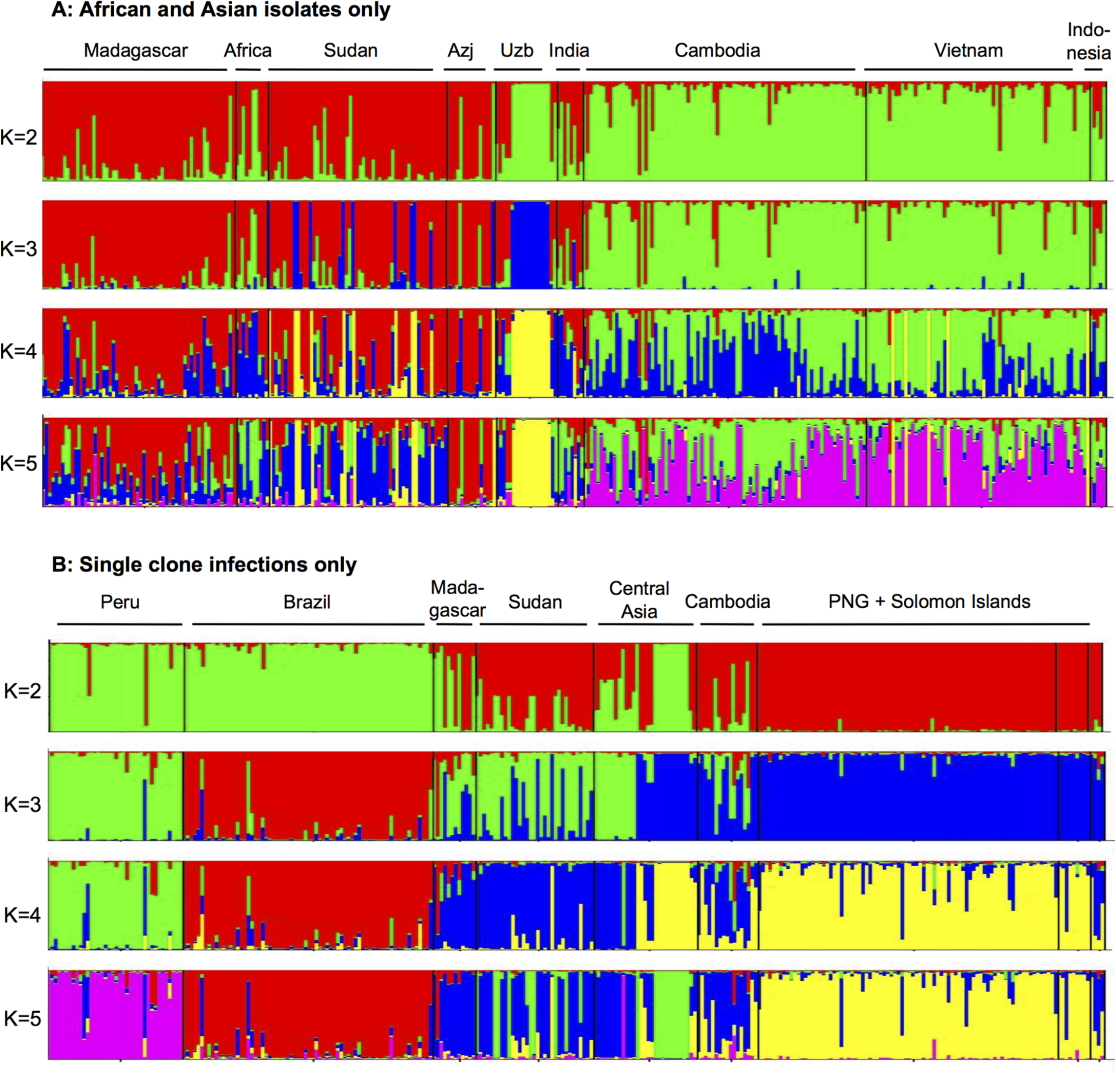
STRUCTURE analysis with African and Asian isolates only (A), and with single clone infections only (B).

As the clear separation of Latin American and South Pacific isolates from those from Africa and Asia might interfere with more subtle population structure within Africa and Asia, clustering analysis by STRUCTURE was repeated including the latter isolates only (Fig 5A). For K = 2 African and Azerbaijani isolates clustered separately from SE-Asian ones. Indian isolates clustered with Africa. As with the full set of isolates, the clonal samples from Uzbekistan clustered with different populations in individual runs for the same number of clusters (e.g. with Africa or with SE-Asia if K = 2). For K = 3 these isolates formed a separate cluster. Both within Africa and SE-Asia admixture was high, and when the number of clusters was set to 4 or 5, the separation between countries was limited. Analysis with STRUCTURE was repeated for single clone infections only. Results were similar as for the full data set (Fig 5B).

Results of clustering analysis were confirmed by *F*_ST_ values (Table 5). Between Brazil and Peru *F*_ST_ values were high (0.16), as well as when South American populations were compared to non-American populations (0.11–0.29). Differences between Cambodia, Vietnam and South Pacific populations were lowest (0.026–0.066). The highest *F*_ST_ value was observed between Azerbaijan and Uzbekistan (0.37), and all values for comparisons between Uzbekistan and other populations were >0.2. *F*_ST_ between Madagascar and Sudan was low (0.07).

**Table 5:** Differentiation of populations (*F*_ST_ values).

	Peru	Brazil	Central America	Madagascar	Sudan	Azerbaijan	Uzbekistan	Vietnam	Cam-bodia	PNG lowlands	PNG highlands
Brazil	0.159										
Central America	0.107	0.095									
Madagascar	0.139	0.125	0.080								
Sudan	0.118	0.142	0.078	0.069							
Azerbaijan	0.208	0.202	0.167	0.140	0.148						
Uzbekistan	0.289	0.279	0.250	0.234	0.246	0.367					
Vietnam	0.109	0.116	0.062	0.071	0.078	0.141	0.206				
Cambodia	0.124	0.136	0.074	0.089	0.096	0.173	0.224	0.032			
PNG lowlands	0.163	0.155	0.097	0.115	0.109	0.154	0.215	0.066	0.055		
PNG highlands	0.160	0.148	0.085	0.110	0.106	0.175	0.237	0.045	0.042	0.026	
Solomon Islands	0.146	0.139	0.096	0.079	0.102	0.165	0.227	0.066	0.058	0.061	0.055

All *F*_ST_ values are significant.

*F*_ST_ values were also compared between continents or sub-continents, i.e. South America, Africa, Central Asia, SE-Asia and the South Pacific (Table 6). *F*_ST_ was highest between South America and the South Pacific as well as between Central Asia and all other populations (0.11), and lowest between SE-Asia and the South Pacific (0.042) and SE-Asia and Africa (0.058).

**Table 6:** *F*_ST_ values among continents.

	South America	Africa	Central Asia	SE-Asia
Africa	0.077			
Central Asia	0.139	0.105		
SE-Asia	0.078	0.058	0.111	
South Pacific	0.111	0.083	0.111	0.042

All *F*_ST_ values are significant.

**Fig 6:**
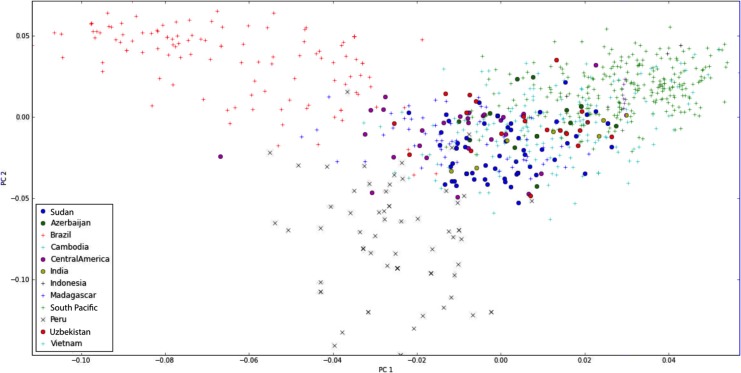
Principal component analysis. Each symbol represents a parasite haplotype, the symbol and color have been assigned according to the geographic origin. Identical haplotypes resulted in the same coordinates in PCA, and are represented by a single symbol; thus the number of symbols plotted does not correspond to the total number of parasite samples analyzed.

In principal component analysis (PCA), PC1 differentiated isolates from Brazil and Peru on the one hand and the South Pacific on the other hand from a cluster containing African and Asian isolates (Fig 6). PC2 separated isolates from Peru und Brazil.

In summary, clustering analysis, *F*_ST_ values and PCA showed similar results. South American populations were separated from all others, and populations from Brazil and Peru formed clearly separated groups with little admixture. African, SE-Asian and South Pacific populations each formed a large cluster with little sub-structuring. Samples from Central America, Indonesia and India grouped with SE-Asia.

## Discussion

Analysis of 841 *P*. *vivax* isolates of global origin, typed with the same markers, allowed direct comparison between populations differing in transmission intensity, geographical isolation and history of malaria control. Determination of the optimal set of microsatellite markers required for differentiation of individual parasite infections will improve strategies for genotyping. Knowledge on population structure can be used to assess the effect of interventions and help to track imported infections.

### Selection of molecular markers for epidemiological studies

Diversity of a marker, expressed as expected heterozygosity (*H*_E_), is a key criterion for choosing a subset of microsatellite markers for a variety of genotyping questions [51]. Markers of a lower diversity are more suitable to assess population structure, as with less diverse markers, a smaller number of isolates is needed to detect genetic differences among populations. In contrast, many applied genotyping studies aim at distinguishing between “identical” and “different” clones. Samples from treatment failures in a drug trial for example require distinction between new infection versus recrudescence (reappearance of a pre-treatment clone) [18–22]. In this scenario the phylogenetic relationship of genotypes observed is not of interest. Such applied typing tasks often include large numbers of isolates, and thus a minimal set of markers is desirable that is able to reduce the genotyping workload and price without impairing the discrimination power for distinguishing clones. For phylogeographical studies a larger panel of markers is needed. The same is required for tracking the origin of imported malaria cases.

The step-wise removal of markers showed that as little as 3 highly diverse markers were sufficient to detect around 90% of all haplotypes in most populations, only in South American populations up to 40% of haplotypes were missed. Thus, typing only 2–3 markers in SE-Asia, the South Pacific and Africa, and 4–6 markers in South America would lead to only a small underestimation of multiplicity of infection or of treatment efficiency (when a clone during follow-up could not be distinguished from the clone at baseline and a new infection was taken for a recrudescence).

Other limitations affect drug trial results, such as imperfect detectability of minority clones [52] and as a consequence substantial day-to-day variation in the alleles detected [53]. Longitudinal *P*. *vivax* studies involving genotyping are also complicated by relapsing hypnozoites, which can be homologous or heterologous to the clone at baseline [16, 54]. Longitudinal studies can also be affected by re-infection with a genotype observed earlier in the same individual. This is a particular threat when the overall diversity in the parasite population is low, as observed in Uzbekistan, or in case of clonal expansion during an outbreak [46]. Thus, when the population diversity is intermediate or high, using a reduced panel of markers is acceptable as this reduces the ability to differentiate clones only in a minor way and is justified in view of substantial savings in time and costs.

### Transmission intensity shapes population parameters

Overall parasite diversity measured as *H*_E_ or allelic richness was high and reflected transmission levels. It was lowest in South America and Central Asia, where transmission is low, and highest in SE-Asia. In the South Pacific diversity was intermediate despite highest levels of transmission (prevalence >50% in Ilaita [19]). This could be due to the relative geographical isolation of South Pacific populations, and thus limited introduction of parasites from other regions. In contrast migration of infected hosts is high among SE-Asian countries, thus high parasite diversity can be maintained, even when transmission is reduced.

Linkage disequilibrium can be the result of selfing in the mosquito of male and female gametes from the same parasite clone [55], as opposed to recombination of different clones resulting in a break up of linkage. Recombination can occur if a mosquito feeds on a host infected with different parasite clones. The level of LD is expected to decrease with increasing transmission intensity, while diversity is expected to increase. In line with this expectation, LD was detected in Brazil, Central Asia and Madagascar, where transmission is low [1]. It is, however, noteworthy that levels of diversity did not fully correlate with transmission intensity. Highest diversity and strong LD was observed in SE-Asia. In the South Pacific an intermediate diversity but no LD was found. Different processes influence diversity and LD; likely the local ecology add to the high parasite diversity in SE-Asia, while the high proportion of individuals carrying multi-clone infections in the South Pacific (up to 75% of all infected [19]) lead to high levels of parasite recombination. High LD is also expected when closely related parasites are sampled. Yet no LD was observed in Ilaita in PNG, despite most dense sampling of all populations with 132 isolates collected across hamlets approx. 5 km from each other [56].

Incorrect assembly of multi-locus haplotypes in multi-clone infections within a host would be expected to lead to incorrect low levels of LD, but the opposite trend was observed: lower LD was found when only single-clone infections were included in the analysis. This unexpected finding was attributed to the smaller sample size after removing multi-clone infections.

Analysis of population structure revealed significant *F*_ST_ values between all populations. While clustering analysis and PCA differentiated among those populations separated by high *F*_ST_ values, no within-continent subdivision was observed in the South Pacific, Africa and SE-Asia. The difference in clustering between populations from Latin American (clearly separated clusters in Peru, Brazil and Central America and Mexico) on the one hand and from the South Pacific (no subdivision between different provinces in PNG and Solomon Islands) or Africa (no subdivision between Madagascar and Sudan) on the other hand is striking, given comparable distances between sites. While high levels of human migration in SE-Asia could explain parasite gene flow, no clusters were found in the South Pacific despite limited human movement (only air and sea transport between East Sepik and Madang provinces, and between PNG and Solomon Islands). Likewise limited sub-structuring was evident between Madagascar and Sudan, despite open sea and countries with very low *P*. *vivax* transmission separating sampling locations. In Central Asia, clear subdivision was observed between parasites east and west of the Caspian Sea. Parasites from Azerbaijan and Armenia clustered with those from Africa, while parasites from Uzbekistan were highly clonal and formed a separate cluster.

Distinct parasite subpopulations might be the result of expansion of parasite clones after the near elimination of malaria in the second half of the 20^th^ century in some countries. In Latin America they could also reflect different independent introductions of *P*. *vivax*, as it has been shown for *P*. *falciparum* [57]. In Uzbekistan *P*. *vivax* had been reintroduced after its elimination, most likely from other Central Asian countries. In contrast, in SE-Asia and the South Pacific *P*. *vivax* was present much longer than in Latin America, and even during the peak of the eradications campaigns in the 1960s prevalence remained high [58]. Central American and Mexican samples were exceptional as they were highly diverse and LD was low despite low transmission intensity. This is most likely caused by the fact that these samples represent different isolated subpopulations over a large geographical range. Further studies involving additional parasite populations and different molecular markers are needed to establish differences between South and Central American samples, to understand why Central America clusters with SE-Asia, and to identify potential routes for *P*. *vivax* colonization of South and Central America.

Beside microsatellites, other molecular markers have been used to assess *P*. *vivax* population structure, most importantly mtDNA and polymorphic antigens. In agreement with the present study, sequencing of mtDNA identified a separate subgroup in Latin America (highest support of all subgroups in Bayesian tree analysis) [36], as well as highly diverse populations in Asia and the South Pacific [37]. However, no pronounced separation between South-Pacific, SE-Asian and some South American isolates was observed using mtDNA [36]. Isolates from South and Central America had been found in the same subgroup, yet, only 3 haplotypes from Central America were sequenced [36]. The same study found different subgroups in East Asia (China and Korea) and SE-Asia (Cambodia, Thailand and Indonesia), plus a third subgroup including isolates from locations across Asia and PNG. The present study includes no isolates from China or Korea, and only few from Indonesia, thus no such structure within East Asia could be found.

In concordance with microsatellite results, mtDNA diversity was highest in SE-Asia and high in the South Pacific [36]. In contrast to microsatellite-derived measurements of diversity, mtDNA diversity was lower for Madagascar, Central America and Africa, whereby results of the latter two populations are likely affected by a very small sample size [36]. The same study indicated that overall *P*. *vivax* diversity in Latin America was as high as in SE-Asia, despite locally reduced diversity, a result confirmed by analysis of 3 whole-genome sequences from Latin America [59]. The present study found microsatellite diversity across Central America and Mexico to be similarly high as that of SE-Asia, but when isolates from Peru and Brazil were combined diversity remained lower.

SNPs in antigens are expected to be under strong balancing selection, limiting their use to study the underlying population structure. In line with this, many antigens showed strong clustering but in contrast to microsatellites many clusters were shared between continents [34, 35]. Like microsatellites, AMA-1 and MSP-1 alleles from South Pacific populations showed very little admixture with any other parasite populations [34, 60]. DBP-II alleles in contrast were more evenly distributed across continents [35]. A study using putatively neutral SNPs covering a 200-kb genomic region confirmed subdivision between Brazil and SE-Asia [61], and a barcode of 42 SNPs across the genome was recently published and tested on a small number of clinical samples from three continents [62]. However, this barcode was not yet tested for assessing local population structure or for typing asymptomatic, low-density infections.

### Application of genotyping in malaria control

Continuous malaria control is expected to reduce parasite diversity and effective population size, and to increase differences between populations due to clonal expansion of remaining parasite strains [63]. Indeed, near-clonal expansion of parasites has been observed for *P*. *falciparum* in the highlands of PNG [8], in Solomon Islands [64] and in South America [7]. Likewise, Artemisinin-resistant clones have expanded in SE-Asia [65].

In striking contrast to these findings, nearly all studies assessing *P*. *vivax* diversity found high parasite diversity, even in countries now aiming to eliminate malaria [66–69]. The clonal expansion in Uzbekistan, a country that had successfully eliminated malaria in the 1960-ies, is the first such population structure reported for *P*. *vivax*. Low microsatellite diversity was also found in South Korea, where transmission has been low for decades and the parasite population is relatively isolated [70], as well as from a rural, isolated site in Peru [71]. The high *P*. *vivax* diversity in countries with low transmission likely indicates a high underlying effective population size and thus a large number of infected individuals. Two hallmarks of *P*. *vivax* biology add to this, namely hypnozoites in the liver, and a large proportion of asymptomatic, low-density infections that escape screenings conducted by light microscopy or rapid diagnostic test and thus a substantially underestimated parasite reservoir [4].

The isolates studied here were collected prior to the renewed call for malaria elimination. Only few studies have typed samples collected after up-scaling control measures, but diversity remained high [72–74]. It seems that control has little short-term effect on population size, and diversity measures changes slowly as long as the effective population size remains high (above 100 genetically distinct parasite clones) [75]. Therefore diversity measures will only be useful to assess the impact of control programs once transmission is very low after several years of intensified control.

In recent years malaria control has been intensified reducing prevalence and incidence in PNG [76], many Asian countries [69, 77] and South America [78]. It will be important to evaluate whether reduced prevalence is paralleled by increased sub-structuring on small scale, i.e. breaking up of the South Pacific and SE-Asian clusters, indicating local hotspots of transmission. A pronounced reduction of genetic diversity and increase in population structure will implicate success of control and interruption of parasite gene flow from neighboring populations.

In previously malaria-free regions, microsatellite typing can help to study outbreaks. Because of their high discrimination power between clones, genotyping outbreak samples can clarify whether a single clone was imported and spread across a local region, or whether steady gene flow from neighboring regions with ongoing transmission occurs, resulting in a diverse parasite population [9].

### Conclusions

Microsatellite typing remains an important tool to study *P*. *vivax*, as it can be done in any lab equipped for PCR. For epidemiological studies and drug trials, a limited set of 2–6 markers, depending on transmission intensity, provides sufficient resolution to distinguish individual clones. The full panel of 11 microsatellite markers showed clear population structure on a global scale, and differences in diversity reflect transmission intensity and isolation of parasite populations. These population genetic measures could potentially be used as tools to measure the impact of control programs; however, due to the large effective population size even in countries of moderate endemicity, these parameters are likely to change slowly.

## Supporting Information

S1 File Measures of diversity by population. Three measures of parasite diversity are listed by marker and population: expected heterozygosity (*H*_*E*_), number of alleles sampled and allelic richness.
(XLSX)

## References

[1] Gething PW, Elyazar IR, Moyes CL, Smith DL, Battle KE, Guerra CA, et al A long neglected world malaria map: *Plasmodium vivax* endemicity in 2010. PLoS Negl Trop Dis. 2012;6(9):e1814 10.1371/journal.pntd.0001814 22970336 PMC3435256

[2] Hay SI, Guerra CA, Tatem AJ, Noor AM, Snow RW. The global distribution and population at risk of malaria: past, present, and future. Lancet Infect Dis. 2004;4(6):327–36. Epub 2004/06/03. 15172341 10.1016/S1473-3099(04)01043-6 PMC3145123

[3] Bousema T, Okell L, Felger I, Drakeley C. Asymptomatic malaria infections: detectability, transmissibility and public health relevance. Nat Rev Microbiol. 2014;12(12):833–40. 10.1038/nrmicro3364 25329408

[4] Baird KJ, Maguire JD, Price RN. Diagnosis and treatment of *Plasmodium vivax* malaria. Advances in parasitology. 2012;80:203–70. 10.1016/B978-0-12-397900-1.00004-9 23199489

[5] Oliveira-Ferreira J, Lacerda MV, Brasil P, Ladislau JL, Tauil PL, Daniel-Ribeiro CT. Malaria in Brazil: an overview. Malar J. 2010;9:115 10.1186/1475-2875-9-115 20433744 PMC2891813

[6] Abeyasinghe RR, Galappaththy GN, Smith Gueye C, Kahn JG, Feachem RG. Malaria control and elimination in Sri Lanka: documenting progress and success factors in a conflict setting. PLoS One. 2012;7(8):e43162 10.1371/journal.pone.0043162 22952642 PMC3430652

[7] Griffing SM, Mixson-Hayden T, Sridaran S, Alam MT, McCollum AM, Cabezas C, et al South American *Plasmodium falciparum* after the malaria eradication era: clonal population expansion and survival of the fittest hybrids. PLoS ONE. 2011;6(9):e23486 Epub 2011/09/29. 10.1371/journal.pone.0023486 21949680 PMC3174945

[8] Mueller I, Kaiok J, Reeder JC, Cortes A. The population structure of *Plasmodium falciparum* and *Plasmodium vivax* during an epidemic of malaria in the Eastern Highlands of Papua New Guinea. Am J Trop Med Hyg. 2002;67(5):459–64. Epub 2002/12/14. 12479544 10.4269/ajtmh.2002.67.459

[9] Ferreira MU, Rodrigues PT. Tracking malaria parasites in the eradication era. Trends Parasitol. 2014;30(10):465–6. 10.1016/j.pt.2014.08.003 25154542

[10] Van den Eede P, Van der Auwera G, Delgado C, Huyse T, Soto-Calle VE, Gamboa D, et al Multilocus genotyping reveals high heterogeneity and strong local population structure of the *Plasmodium vivax* population in the Peruvian Amazon. Malar J. 2010;9(1):151. 20525233 10.1186/1475-2875-9-151 PMC2898784

[11] Koepfli C, Timinao L, Antao T, Barry AE, Siba P, Mueller I, et al A Large *Plasmodium vivax* Reservoir and Little Population Structure in the South Pacific. PLoS One. 2013;8(6):e66041 23823758 10.1371/journal.pone.0066041 PMC3688846

[12] Gunawardena S, Karunaweera ND, Ferreira MU, Phone-Kyaw M, Pollack RJ, Alifrangis M, et al Geographic structure of *Plasmodium vivax*: microsatellite analysis of parasite populations from Sri Lanka, Myanmar, and Ethiopia. Am J Trop Med Hyg. 2010;82(2):235–42. Epub 2010/02/06. 10.4269/ajtmh.2010.09-0588 20133999 PMC2813164

[13] Imwong M, Nair S, Pukrittayakamee S, Sudimack D, Williams JT, Mayxay M, et al Contrasting genetic structure in *Plasmodium vivax* populations from Asia and South America. Int J Parasitol. 2007;37(8–9):1013–22. 17442318 10.1016/j.ijpara.2007.02.010

[14] Ferreira MU, Karunaweera ND, da Silva-Nunes M, da Silva NS, Wirth DF, Hartl DL. Population structure and transmission dynamics of *Plasmodium vivax* in rural Amazonia. J Infect Dis. 2007;195(8):1218–26. Epub 2007/03/16. 17357061 10.1086/512685

[15] Imwong M, Boel ME, Pagornrat W, Pimanpanarak M, McGready R, Day NP, et al The first *Plasmodium vivax* relapses of life are usually genetically homologous. J Infect Dis. 2012;205(4):680–3. 10.1093/infdis/jir806 22194628 PMC3266132

[16] Imwong M, Snounou G, Pukrittayakamee S, Tanomsing N, Kim JR, Nandy A, et al Relapses of *Plasmodium vivax* infection usually result from activation of heterologous hypnozoites. J Infect Dis. 2007;195(7):927–33. 17330781 10.1086/512241

[17] Restrepo E, Imwong M, Rojas W, Carmona-Fonseca J, Maestre A. High genetic polymorphism of relapsing *P*. *vivax* isolates in northwest Colombia. Acta Trop. 2011;119(1):23–9. Epub 2011/04/19. 10.1016/j.actatropica.2011.03.012 21497586 PMC3485554

[18] Koepfli C, Colborn KL, Kiniboro B, Lin E, Speed TP, Siba PM, et al A High Force of *Plasmodium vivax* Blood-Stage Infection Drives the Rapid Acquisition of Immunity in Papua New Guinean Children. PLoS Negl Trop Dis. 2013;7(9):e2403 10.1371/journal.pntd.0002403 24040428 PMC3764149

[19] Koepfli C, Ross A, Kiniboro B, Smith TA, Zimmerman PA, Siba P, et al Multiplicity and Diversity of *Plasmodium vivax* Infections in a Highly Endemic Region in Papua New Guinea. PLoS Negl Trop Dis. 2011;5(12):e1424 Epub 2011/12/30. 10.1371/journal.pntd.0001424 22206027 PMC3243695

[20] Barnadas C, Koepfli C, Karunajeewa HA, Siba PM, Davis TM, Mueller I. Characterization of treatment failure in efficacy trials of drugs against *Plasmodium vivax* by genotyping neutral and drug resistance-associated markers. Antimicrob Agents Chemother. 2011;55(9):4479–81. Epub 2011/06/29. 10.1128/AAC.01552-10 21709097 PMC3165340

[21] Orjuela-Sanchez P, da Silva NS, da Silva-Nunes M, Ferreira MU. Recurrent parasitemias and population dynamics of *Plasmodium vivax* polymorphisms in rural Amazonia. Am J Trop Med Hyg. 2009;81(6):961–8. Epub 2009/12/10. 10.4269/ajtmh.2009.09-0337 19996423

[22] Hwang J, Alemayehu BH, Reithinger R, Tekleyohannes SG, Takele T, Birhanu SG, et al In vivo efficacy of artemether-lumefantrine and chloroquine against *Plasmodium vivax*: a randomized open label trial in central Ethiopia. PLoS One. 2013;8(5):e63433 10.1371/journal.pone.0063433 23717423 PMC3661577

[23] Jennison C, Arnott A, Tessier N, Tavul L, Koepfli C, Felger I, et al *Plasmodium vivax* populations are more genetically diverse and less structured than sympatric *Plasmodium falciparum* populations. PLoS Negl Trop Dis. 2015;9(4):e0003634 10.1371/journal.pntd.0003634 25874894 PMC4398418

[24] Sutton PL. A call to arms: on refining Plasmodium vivax microsatellite marker panels for comparing global diversity. Malar J. 2013;12(1):447. Epub 2013/12/18. 24330329 10.1186/1475-2875-12-447 PMC3878832

[25] Menegon M, Durand P, Menard D, Legrand E, Picot S, Nour B, et al Genetic diversity and population structure of *Plasmodium vivax* isolates from Sudan, Madagascar, French Guiana and Armenia. Infect Genet Evol. 2014;27:244–9. 10.1016/j.meegid.2014.07.029 25102032

[26] Orjuela-Sanchez P, Sa JM, Brandi MC, Rodrigues PT, Bastos MS, Amaratunga C, et al Higher microsatellite diversity in *Plasmodium vivax* than in sympatric *Plasmodium falciparum* populations in Pursat, Western Cambodia. Exp Parasitol. 2013;134(3):318–26. 10.1016/j.exppara.2013.03.029 23562882 PMC3691688

[27] Van den Eede P, Erhart A, Van der Auwera G, Van Overmeir C, Thang ND, Hung le X, et al High complexity of *Plasmodium vivax* infections in symptomatic patients from a rural community in central Vietnam detected by microsatellite genotyping. Am J Trop Med Hyg. 2010;82(2):223–7. Epub 2010/02/06. 10.4269/ajtmh.2010.09-0458 20133996 PMC2813161

[28] Karunaweera ND, Ferreira M. U., Hartl D. L., Wirth D. F. Fourteen polymorphic microsatellite DNA markers for the human malaria parasite *Plasmodium vivax* . Molecular Ecology Notes. 2006;7(1):172–5.

[29] Battle KE, Karhunen MS, Bhatt S, Gething PW, Howes RE, Golding N, et al Geographical variation in *Plasmodium vivax* relapse. Malar J. 2014;13:144 10.1186/1475-2875-13-144 24731298 PMC4021508

[30] Erhart A, Ngo DT, Phan VK, Ta TT, Van Overmeir C, Speybroeck N, et al Epidemiology of forest malaria in central Vietnam: a large scale cross-sectional survey. Malar J. 2005;4:58 16336671 10.1186/1475-2875-4-58 PMC1325238

[31] Joy DA, Gonzalez-Ceron L, Carlton JM, Gueye A, Fay M, McCutchan TF, et al Local adaptation and vector-mediated population structure in *Plasmodium vivax* malaria. Mol Biol Evol. 2008;25(6):1245–52. 10.1093/molbev/msn073 18385220 PMC2386084

[32] Howes RE, Patil AP, Piel FB, Nyangiri OA, Kabaria CW, Gething PW, et al The global distribution of the Duffy blood group. Nat Commun. 2011;2:266 10.1038/ncomms1265 21468018 PMC3074097

[33] Razakov Sh A, Shakhgunova G. [Current malaria situation in the Republic of Uzbekistan]. Med Parazitol (Mosk). 2001;(1):39–41. 11548313

[34] Arnott A, Mueller I, Ramsland PA, Siba PM, Reeder JC, Barry AE. Global Population Structure of the Genes Encoding the Malaria Vaccine Candidate, *Plasmodium vivax* Apical Membrane Antigen 1 (PvAMA1). PLoS Negl Trop Dis. 2013;7(10):e2506 10.1371/journal.pntd.0002506 24205419 PMC3814406

[35] Nobrega de Sousa T, Carvalho LH, Alves de Brito CF. Worldwide genetic variability of the Duffy binding protein: insights into *Plasmodium vivax* vaccine development. PLoS One. 2011;6(8):e22944 10.1371/journal.pone.0022944 21829672 PMC3149059

[36] Taylor JE, Pacheco MA, Bacon DJ, Beg MA, Machado RL, Fairhurst RM, et al The evolutionary history of *Plasmodium vivax* as inferred from mitochondrial genomes: parasite genetic diversity in the Americas. Mol Biol Evol. 2013;30(9):2050–64. 10.1093/molbev/mst104 23733143 PMC3748350

[37] Mu J, Joy DA, Duan J, Huang Y, Carlton J, Walker J, et al Host switch leads to emergence of *Plasmodium vivax* malaria in humans. Mol Biol Evol. 2005;22(8):1686–93. 15858201 10.1093/molbev/msi160

[38] Rodrigues PT, Alves JM, Santamaria AM, Calzada JE, Xayavong M, Parise M, et al Using mitochondrial genome sequences to track the origin of imported *Plasmodium vivax* infections diagnosed in the United States. Am J Trop Med Hyg. 2014;90(6):1102–8. 10.4269/ajtmh.13-0588 24639297 PMC4047736

[39] Antao T, Lopes A, Lopes RJ, Beja-Pereira A, Luikart G. LOSITAN: A workbench to detect molecular adaptation based on a F(st)-outlier method. BMC Bioinformatics. 2008;9. 10.1186/1471-2105-9-323 PMC2515854 18662398

[40] Matschiner M, Salzburger W. TANDEM: integrating automated allele binning into genetics and genomics workflows. Bioinformatics. 2009;25(15):1982–3. Epub 2009/05/08. 10.1093/bioinformatics/btp303 19420055

[41] Lischer HE, Excoffier L. PGDSpider: an automated data conversion tool for connecting population genetics and genomics programs. Bioinformatics. 2012;28(2):298–9. 10.1093/bioinformatics/btr642 22110245

[42] Goudet J. FSTAT (vers. 1.2): a computer program to calculate F-statistics. Journal of Heredity. 1995;86:485–6.

[43] McKelvey KS, Schwartz MK. DROPOUT: a program to identify problem loci and samples for noninvasive genetic samples in a capture-mark-recapture framework. Molecular Ecology Notes. 2005;5(3):716–8.

[44] Haubold B, Hudson RR. LIAN 3.0: detecting linkage disequilibrium in multilocus data. Linkage Analysis. Bioinformatics. 2000;16(9):847–8. Epub 2000/12/08. 11108709 10.1093/bioinformatics/16.9.847

[45] Anderson TJ, Su XZ, Roddam A, Day KP. Complex mutations in a high proportion of microsatellite loci from the protozoan parasite *Plasmodium falciparum* . Mol Ecol. 2000;9(10):1599–608. Epub 2000/10/26. 11050555 10.1046/j.1365-294x.2000.01057.x

[46] Batista CL, Barbosa S, Da Silva Bastos M, Viana SA, Ferreira MU. Genetic diversity of *Plasmodium vivax* over time and space: a community-based study in rural Amazonia. Parasitology. 2015;142(2):374–84. 10.1017/S0031182014001176 25068581

[47] Pritchard JK, Stephens M, Donnelly P. Inference of population structure using multilocus genotype data. Genetics. 2000;155(2):945–59. Epub 2000/06/03. 10835412 10.1093/genetics/155.2.945 PMC1461096

[48] Evanno G, Regnaut S, Goudet J. Detecting the number of clusters of individuals using the software STRUCTURE: a simulation study. Mol Ecol. 2005;14(8):2611–20. Epub 2005/06/23. 15969739 10.1111/j.1365-294X.2005.02553.x

[49] Huelsenbeck JP, Andolfatto P, Huelsenbeck ET. Structurama: bayesian inference of population structure. Evol Bioinform Online. 2011;7:55–9. 10.4137/EBO.S6761 21698091 PMC3118697

[50] Patterson N, Price AL, Reich D. Population structure and eigenanalysis. PLoS genetics. 2006;2(12):e190 17194218 10.1371/journal.pgen.0020190 PMC1713260

[51] Chenet SM, Schneider KA, Villegas L, Escalante AA. Local population structure of *Plasmodium*: impact on malaria control and elimination. Malar J. 2012;11:412 10.1186/1475-2875-11-412 23232077 PMC3538601

[52] Juliano JJ, Gadalla N, Sutherland CJ, Meshnick SR. The perils of PCR: can we accurately 'correct' antimalarial trials? Trends Parasitol. 2010;26(3):119–24. Epub 2010/01/20. 10.1016/j.pt.2009.12.007 20083436 PMC2844636

[53] Koepfli C, Schoepflin S, Bretscher M, Lin E, Kiniboro B, Zimmerman PA, et al How Much Remains Undetected? Probability of Molecular Detection of Human *Plasmodia* in the Field. PLoS ONE. 2011;6(4):e19010 Epub 2011/05/10. 10.1371/journal.pone.0019010 21552561 PMC3084249

[54] de Araujo FC, de Rezende AM, Fontes CJ, Carvalho LH, Alves de Brito CF. Multiple-clone activation of hypnozoites is the leading cause of relapse in *Plasmodium vivax* infection. PLoS One. 2012;7(11):e49871 10.1371/journal.pone.0049871 23185469 PMC3503861

[55] Mzilahowa T, McCall PJ, Hastings IM. "Sexual" population structure and genetics of the malaria agent *P*. *falciparum* . PLoS ONE. 2007;2(7):e613 Epub 2007/07/20. 17637829 10.1371/journal.pone.0000613 PMC1910609

[56] Lin E, Kiniboro B, Gray L, Dobbie S, Robinson L, Laumaea A, et al Differential patterns of infection and disease with *P*. *falciparum* and *P*. *vivax* in young Papua New Guinean children. PLoS ONE. 2010;5(2):e9047 Epub 2010/02/09. 10.1371/journal.pone.0009047 20140220 PMC2816213

[57] Yalcindag E, Elguero E, Arnathau C, Durand P, Akiana J, Anderson TJ, et al Multiple independent introductions of *Plasmodium falciparum* in South America. Proc Natl Acad Sci U S A. 2012;109(2):511–6. 10.1073/pnas.1119058109 22203975 PMC3258587

[58] Muller I, Bockarie M, Alpers M, Smith T. The epidemiology of malaria in Papua New Guinea. Trends Parasitol. 2003;19(6):253–9. 12798082 10.1016/s1471-4922(03)00091-6

[59] Neafsey DE, Galinsky K, Jiang RH, Young L, Sykes SM, Saif S, et al The malaria parasite *Plasmodium vivax* exhibits greater genetic diversity than *Plasmodium falciparum* . Nat Genet. 2012;44(9):1046–50. Epub 2012/08/07. 10.1038/ng.2373 22863733 PMC3432710

[60] Figtree M, Pasay CJ, Slade R, Cheng Q, Cloonan N, Walker J, et al *Plasmodium vivax* synonymous substitution frequencies, evolution and population structure deduced from diversity in AMA 1 and MSP 1 genes. Mol Biochem Parasitol. 2000;108(1):53–66. Epub 2000/05/10. 10802318 10.1016/s0166-6851(00)00204-8

[61] Orjuela-Sanchez P, Karunaweera ND, da Silva-Nunes M, da Silva NS, Scopel KK, Goncalves RM, et al Single-nucleotide polymorphism, linkage disequilibrium and geographic structure in the malaria parasite *Plasmodium vivax*: prospects for genome-wide association studies. BMC genetics. 2010;11:65 10.1186/1471-2156-11-65 20626846 PMC2910014

[62] Baniecki ML, Faust AL, Schaffner SF, Park DJ, Galinsky K, Daniels RF, et al Development of a single nucleotide polymorphism barcode to genotype *Plasmodium vivax* infections. PLoS Negl Trop Dis. 2015;9(3):e0003539 10.1371/journal.pntd.0003539 25781890 PMC4362761

[63] Volkman SK, Neafsey DE, Schaffner SF, Park DJ, Wirth DF. Harnessing genomics and genome biology to understand malaria biology. Nat Rev Genet. 2012;13(5):315–28. 10.1038/nrg3187 22495435

[64] Ballif M, Hii J, Marfurt J, Crameri A, Fafale A, Felger I, et al Monitoring of malaria parasite resistance to chloroquine and sulphadoxine-pyrimethamine in the Solomon Islands by DNA microarray technology. Malar J. 2010;9:270 Epub 2010/10/12. 10.1186/1475-2875-9-270 20925934 PMC2959069

[65] Miotto O, Almagro-Garcia J, Manske M, Macinnis B, Campino S, Rockett KA, et al Multiple populations of artemisinin-resistant *Plasmodium falciparum* in Cambodia. Nat Genet. 2013;45(6):648–55. 10.1038/ng.2624 23624527 PMC3807790

[66] Gonzalez-Ceron L, Mu J, Santillan F, Joy D, Sandoval MA, Camas G, et al Molecular and epidemiological characterization of *Plasmodium vivax* recurrent infections in southern Mexico. Parasites & vectors. 2013;6:109. 23597046 10.1186/1756-3305-6-109 PMC3637411

[67] Gray KA, Dowd S, Bain L, Bobogare A, Wini L, Shanks GD, et al Population genetics of *Plasmodium falciparum* and *Plasmodium vivax* and asymptomatic malaria in Temotu Province, Solomon Islands. Malar J. 2013;12:429 10.1186/1475-2875-12-429 24261646 PMC4222835

[68] Leclerc MC, Menegon M, Cligny A, Noyer JL, Mammadov S, Aliyev N, et al Genetic diversity of *Plasmodium vivax* isolates from Azerbaijan. Malar J. 2004;3:40 15535878 10.1186/1475-2875-3-40 PMC534801

[69] Gunawardena S, Ferreira MU, Kapilananda GM, Wirth DF, Karunaweera ND. The Sri Lankan paradox: high genetic diversity in *Plasmodium vivax* populations despite decreasing levels of malaria transmission. Parasitology. 2014;141(7):880–90. 10.1017/S0031182013002278 24533989 PMC7485621

[70] Iwagami M, Fukumoto M, Hwang SY, Kim SH, Kho WG, Kano S. Population structure and transmission dynamics of *Plasmodium vivax* in the Republic of Korea based on microsatellite DNA analysis. PLoS Negl Trop Dis. 2012;6(4):e1592 10.1371/journal.pntd.0001592 22509416 PMC3317904

[71] Delgado-Ratto C, Soto-Calle VE, Van den Eede P, Gamboa D, Rosas A, Abatih EN, et al Population structure and spatio-temporal transmission dynamics of *Plasmodium vivax* after radical cure treatment in a rural village of the Peruvian Amazon. Malar J. 2014;13:8 10.1186/1475-2875-13-8 24393454 PMC3893378

[72] Abdullah NR, Barber BE, William T, Norahmad NA, Satsu UR, Muniandy PK, et al *Plasmodium vivax* population structure and transmission dynamics in Sabah Malaysia. PLoS One. 2013;8(12):e82553 10.1371/journal.pone.0082553 24358203 PMC3866266

[73] Liu Y, Auburn S, Cao J, Trimarsanto H, Zhou H, Gray KA, et al Genetic diversity and population structure of *Plasmodium vivax* in Central China. Malar J. 2014;13:262 10.1186/1475-2875-13-262 25008859 PMC4094906

[74] Noviyanti R, Coutrier F, Utami RA, Trimarsanto H, Tirta YK, Trianty L, et al Contrasting Transmission Dynamics of Co-endemic *Plasmodium vivax* and *P*. *falciparum*: Implications for Malaria Control and Elimination. PLoS Negl Trop Dis. 2015;9(5):e0003739 10.1371/journal.pntd.0003739 25951184 PMC4423885

[75] Nkhoma SC, Nair S, Al-Saai S, Ashley E, McGready R, Phyo AP, et al Population genetic correlates of declining transmission in a human pathogen. Mol Ecol. 2013;22(2):273–85. 10.1111/mec.12099 23121253 PMC3537863

[76] Koepfli C, Robinson LJ, Rarau P, Salib M, Sambale N, Wampfler R, et al Blood-Stage Parasitaemia and Age Determine *Plasmodium falciparum* and *P*. *vivax* Gametocytaemia in Papua New Guinea. PLoS One. 2015;10(5):e0126747 10.1371/journal.pone.0126747 25996916 PMC4440770

[77] Bhatia R, Rastogi RM, Ortega L. Malaria successes and challenges in Asia. Journal of vector borne diseases. 2013;50(4):239–47. 24499845

[78] Batista CL, Barbosa S, M DASB, Viana SA, Ferreira MU. Genetic diversity of Plasmodium vivax over time and space: a community-based study in rural Amazonia. Parasitology. 2014:1–11. 10.1017/S0031182014001176 25068581

[79] Barnadas C, Ratsimbasoa A, Tichit M, Bouchier C, Jahevitra M, Picot S, et al *Plasmodium vivax* resistance to chloroquine in Madagascar: clinical efficacy and polymorphisms in pvmdr1 and pvcrt-o genes. Antimicrob Agents Chemother. 2008;52(12):4233–40. 10.1128/AAC.00578-08 18809933 PMC2592859

